# Supplementary material for: Modulation of T Regulatory and Dendritic Cell Phenotypes Following Ingestion of Bifidobacterium longum, AHCC® and Azithromycin in Healthy Individuals
Source: Nutrients. 2019 Oct 15;11(10):2470. doi: 10.3390/nu11102470 (PMC6835407; doi:10.3390/nu11102470)
Supplement: Supplementary file 1 [file nutrients-11-02470-s001.pdf]

SUPPLEMENTARY METHODS

1 Collection of Human Peripheral Blood Polymorphonuclear Cells (PBMCs)

Whole blood from volunteers was collected into glass vials containing 170 IU of lithium heparin per 10 ml of blood (Vacutainer™, Becton Dickinson, Cowley, and Oxford, UK). This was then diluted with an equal volume of phosphate buffered saline (PBS) (Oxoid™, Thermo Scientific, Hampshire, UK) and 10 ml then carefully layered over 4ml of Histopaque®1077 (Sigma-Aldrich, Gillingham, UK), in a 15 ml conical polypropylene centrifuge tube (Cellstar®, Griener Bio-One, Frickenhausen, Germany). Tubes were then centrifuged at 400 × g for 30 min at 37°C, without braking in an Eppendorf 5702R centrifuge (Eppendorf AG, Hamburg, Germany). After centrifugation the supernatant was aspirated to within 0.5 cm of the liquid interface and the mononuclear cells then aspirated from the interface into a clean conical centrifuge tube. The cells were washed twice in 10 ml of Hank's balanced salt solution (Sigma-Aldrich, UK) and centrifuged at 250 × g for 10 min at room temperature (RT). Following the second wash the cells were counted using a Brightline® Haemacytometer (Sigma-Aldrich, UK). PBMCs were resuspended in 4°C freezing media consisting of 90% foetal calf serum (FCS) plus 10% dimethyl sulfoxide (Sigma-Aldrich, UK) at 10^6 cells/ml. 1 ml volumes of cell suspensions were placed in 1.8 ml cryovials (Wheaton Cryule Vials; Fisher Scientific, Pittsburgh, PA, USA). Cryovials were labelled and placed into the freezing chamber of a freezing container ('Mr. Frosty' Thermo Scientific, Nalgene, Rochester, NY, USA) for a controlled freezing rate of 1°C per min from 4° to −80°C. Cryovials were stored at −80°C.

2 Cell culture Medium

Human PBMCs, human T-lymphocytes and human monocytes were maintained in complete cell culture medium (CTCM) consisting of endotoxin-free Roswell Park Memorial Institute (RPMI) 1640 supplemented with 10% human AB serum (HALB, Sigma-Aldrich, UK), 100 U/ml penicillin/100 μg/ml streptomycin (Sigma-Aldrich, UK), 2 mM L-glutamine (Sigma-Aldrich, UK) and 10mM 4-(2-hydroxyethyl)-1-piperazineethanesulfonic acid (HEPES) (Sigma-Aldrich, UK).

3 Cell density Determination

Cell density and viability were determined by trypan blue (Sigma-Aldrich, UK) dye exclusion. This method is based on the principle that viable cells are not permeable to certain dyes, whereas non-viable cells are. Cell suspension was mixed thoroughly via pipetting before adding 5 μl of cell suspension to 20 μl of trypan blue solution [0.4% (w/v) trypan blue in PBS]. The trypan blue-cell suspension was transferred to both chambers of a Brightline® Haemocytometer (Sigma-Aldrich, UK). Cell concentration per ml and the total number of cells were determined using the following calculations:

Cells per ml = Total cell count × 10^4 × 5 (dilution factor)

Total cell no. = Cells per ml × original vol. of cell suspension

4 Assay of Cytokine Concentration Using Flow Cytometry

Cytokines at various time points were assayed using flow cytometry and the cytometric bead array technique. The BenderMedSystems multiplex flowcytomix kit (Bender Medystems, Ebioscience, San Diego, CA, USA) was chosen to perform all cytokine assays. This kit employs a series of particles with discrete fluorescence intensities to simultaneously detect multiple soluble analytes, and when combined with flow cytometry, a multiplexed assay is created. The BenderMedSystems Human Th1/Th2 Cytokine Kit (Bender Medystems, USA) was used to quantitatively measure IL-2, IL-4, IL-5, IL-6, IL-10, TNF-α and IFN-γ protein levels in a single sample.

Firstly, the human cytokine standards were prepared. One vial of lyophilized human cytokine standards was reconstituted with 200 μl of assay diluents (provided in kit) to prepare a 10× bulk standard. The reconstituted standard was allowed to equilibrate for 20 min before agitating the vial to mix thoroughly. The 10× bulk standard was aliquoted to 10 μl Eppendorfs and stored at −20°C. 12

× 75 mm fluorescein-activated cell sorter (FACS) tubes (BD Biosciences, San Jose, CA, USA) were labelled and arranged in the following order: Top Standard, 1:2, 1:4, 1:8, 1:16, 1:32, 1:64, 1:128, and 1:256. 90 μl of assay diluents was added to the top standard tube while 30 μl of assay diluents was added to each of the remaining tube. 10 μl of 10× bulk standard was then transferred to the top standard tube and mixed thoroughly by inverting tube.

Serial dilutions followed by transferring 30 μl from the top standard to the 1:2 dilution tube and mixed thoroughly before transferring 30 μl from the 1:2 tube to 1:4 tube and so on to the 1:256 tube mixing thoroughly each time. The assay diluents reagent served as the negative control.

Next, mixed human cytokine capture beads were prepared. The capture beads were bottled individually (i.e. each cytokine in a different bottle) and were pooled together immediately before use. The number of assay tubes including standards and controls was determined and labelled. The capture bead suspension was vigorously vortexed for a few seconds before use. 3 μl aliquot of each capture bead for each assay tube to be analysed was added to a single tube labelled 'mixed capture beads' and vortexed to mix thoroughly. 15 μl of the mixed capture beads was added to the appropriate assay tubes followed by 15 μl of the human PE detection reagent (included in the kit). 15 μl of the human cytokine standard dilutions was then added to the control assay tubes while 15 μl of test samples was added to the test assay tubes. The assay tubes were incubated for 3 h at room temperature and protected from direct exposure to light before washing with 500 μl of wash buffer (included in kit) and centrifuged at 200 × g for 5 min. The supernatant was carefully aspirated and discarded from each assay tube and the bead pellets re-suspended in 300 μl of wash buffer before reading on a flow cytometer. Results were analysed using the Bender MedSystems Flow Cytomix Software Package (Bender Medystems, eBioscience, USA).

5 Purification of $CD3^+$ Cells from PBMCs

Cryopreserved cells, which had been stored for 2 weeks to 18 months, were thawed by placing the cryovials in a water bath at 37°C for 5 min. An equal volume of FBS pre-warmed to 37°C was gently added to the vial. The suspension was allowed to equilibrate in the water bath for 5 min and was then gently layered over 10 ml of RPMI 1640 (Sigma-Aldrich, UK) at 37°C. The cell suspension was allowed to equilibrate for 5 min, during which time the denser DMSO cell suspension settled to the bottom of the centrifuge tube. The tube was gently inverted twice to mix the suspension. After centrifugation for 10 min at 500 × g, cells were re-suspended in PBS plus 10% FCS, counted, and scored for viability by trypan blue exclusion.

6 Immunomagnetic Selection of $CD3^+$ Subpopulations

6.1 Principle of Immunomagnetic Separation

First, $CD3^+$ cells were magnetically labelled with CD3 MicroBeads (Miltenyi Biotec, Auburn, CA, USA). The cell suspension was loaded onto a MACS® Column (Miltenyi Biotec, USA) and placed into the magnetic field of a MACS Separator (Miltenyi Biotec, USA). The magnetically labelled CD3+ cells were retained on the column. The unlabelled cells were run through and this cell fraction was depleted of $CD3^+$ cells. After removal of the column from the magnetic field, the magnetically retained $CD3^+$ cells were eluted as the positively selected cell fraction. To isolate $CD3^+$ T cells, 10^7 PBMCs were mixed with 20 μL of human CD3+ cell isolation MACS microbeads (Miltenyi Biotec, USA) in a total volume of 80 μL and incubated on ice for 30 minutes. During incubation an LS column (Miltenyi Biotec, USA) was primed by washing twice with MACS buffer (Miltenyi Biotec, USA). On completion of the incubation period, the MACS microbeads labelled cell suspension was loaded onto the LS column followed by washing twice with MACS buffer. On removal from the magnetic field $CD3^+$ T cells retained in the column were eluted as the positively selected fraction. This fraction was then washed in MACS buffer twice and then resuspended in RPMI with glutamine and BSA.

6.2 Stimulation of Cytokine Release from $CD3^+$ Cells

For activation, $CD3^+$ cells were resuspended at a concentration of 4×10^5 cells per well in complete culture medium, e.g. RPMI 1640 supplemented with 2 mM L-glutamine, penicillin, streptomycin, 20% human serum and of phorbol 12,13-dibutyrate (PDB) (10 nM)/ionomycin (0.5 μM). 100 μl were distributed per well in duplicate. Complete culture medium was then added to bring the final volume to 200 μl. The plates were then incubated for 24 h at 37°C prior to centrifugation at 5000 × g for 15 min to collect the supernatants. These were placed into marked cryovials and snap frozen in liquid nitrogen before storage at −80°C.

6.3 Flow Cytometric Determination of Foxp3 Expression

PBMCs were stained for cell surface markers for 30 minutes with 2.5 μl phycoerythrin Texas red conjugate [energy couple dye (ECD)] antihuman CD4 (eBioscience, San Diego, CA, USA) and 5μl phycoerythrin (PE) antihuman CD25 (eBioscience, USA). Cell surface markers for CD4 and CD25 were determined by staining for 30 minutes with 2.5 μl of ECD antihuman CD4 and 5μl fluorescein isothiocyanate (FITC) antihuman CD25 (eBioscience, USA). The cells were then washed with RPMI and 2% FCS; 2% formaldehyde was used for fixation of PBMCs for 10 minutes at room temperature (RT). The PBMCs were then washed once in phosphate buffered saline (PBS) containing 2% FCS, twice in PBS/0.5% Tween with 0.05% azide and 3% FCS. 2.5μl FITC antihuman Foxp3 (intracellular) was added to the corresponding tubes and incubated for 2 hours at 4°C (shaking gently every 20 min).

The PBMC pellet was then washed twice in PBS/0.5% Tween, 0.05% azide and 3% FCS. The pellet was then resuspended in 400 μl of 0.5% paraformaldehyde fixative solution for FC analysis.

The BD™CompBeads (BD Biosciences, USA) anti-mouse Ig, κ polystyrene microparticles were used to optimise fluorescence compensation settings for multicolour flow cytometric analyses. The set provided two populations of microparticles, the BD™CompBeads anti-mouse Ig, κ particles, which bind any mouse κ light chain-bearing immunoglobulin, and the BD™CompBeads negative control (FCS), which has no binding capacity. When mixed together with a fluorochrome-conjugated mouse antibody, the BD™CompBeads provide distinct positive and negative (background fluorescence) stained populations which can be used to set compensation levels manually or using instrument set-up software.

For each flow cytometric analysis sample an appropriate corresponding tube was set up with a minus fluorescence control (to identify and eliminate all possible non-specific antibody binding and to ensure accurate data acquisition and interpretation was carried out).

For the analysis, lymphocytes were gated from total PBMCs. Following this, a gate was created from cells demonstrating $CD4^{high}$ SS^{low}. Subsequent gate was created to select cells demonstrating $CD4^{high}$ $Foxp3^{high}$. A final gate selecting cells with $CD25^{high}$ $Foxp3^{high}$ allowed the assay of $CD4^+CD25^+$ $Foxp3^+$ cells (Figure S1).

Peripheral blood DCs can be divided specific phenotypic subsets based on the expression of CD11c. Furthermore, $CD11c^+$ DC can be subdivided into at least two other distinct phenotypes: a major $CD1c^+$ subset and a minor $CD1c^-$ subset [1]. Further definition and characterisation of peripheral blood DC subsets have been facilitated by identification of subset-specific surface markers CD141 (BDCA-3), CD303 (BDCA-2), and CD304 (BDCA-4).

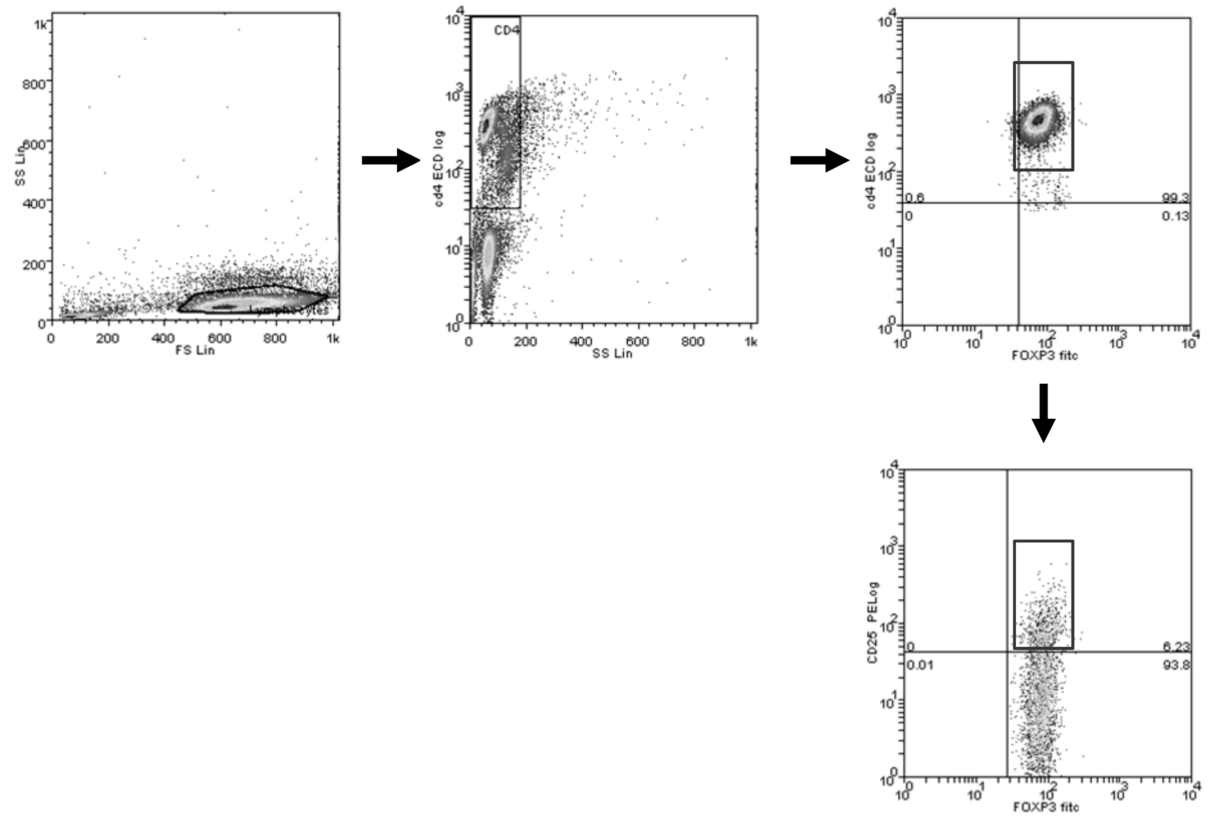

Figure S1. Gating strategy for the detection of Foxp3 from $CD4^{+}$ $CD25^{+}$ T cells. Lymphocytes are selected on a dot-plot of cells with high forward scatter and low side scatter. Cells demonstrating $CD4^{high}SS^{low}$ are selected. Further gates of $CD4^{high}Foxp3^{high}$ and $CD25^{high}Foxp3^{high}$ are applied to derive $CD4^{+}CD25^{+}Foxp3^{+}$ cells. SS: side scatter; FS: forward scatter; lin: linear; ECD: energy couple dye; PE: phycoerythrin, FITC: Fluorescein isothiocyanate.

7 Detection of Dendritic Cell Subsets by Flow Cytometry.

In the absence of pathogenic stimuli, CD303 and CD304 are co-expressed on $CD11c^{-}$ pDCs, whereas both $CD1c^{+}$ and $CD141^{+}$ markers are associated with $CD11c^{+}$ mDCs. These markers can, therefore, be used to further define mDC subsets, $CD11c^{+}CD1c^{+}CD141^{-}$ (mDC1) and $CD11c^{+}CD1c^{-}CD141^{+}$ (mDC2), respectively. Importantly, there is no CD1c or CD141 expression on pDC, which can be used to differentiate mDC and pDC phenotypes [1]. Under steady state conditions, both mDC and pDC exhibit immature phenotypes with low expression of MHC molecules and the costimulatory molecules CD40, CD80, and CD86 [1–3]. A scheme of surface markers identification for mDCs and pDCs is shown in Figure S2.

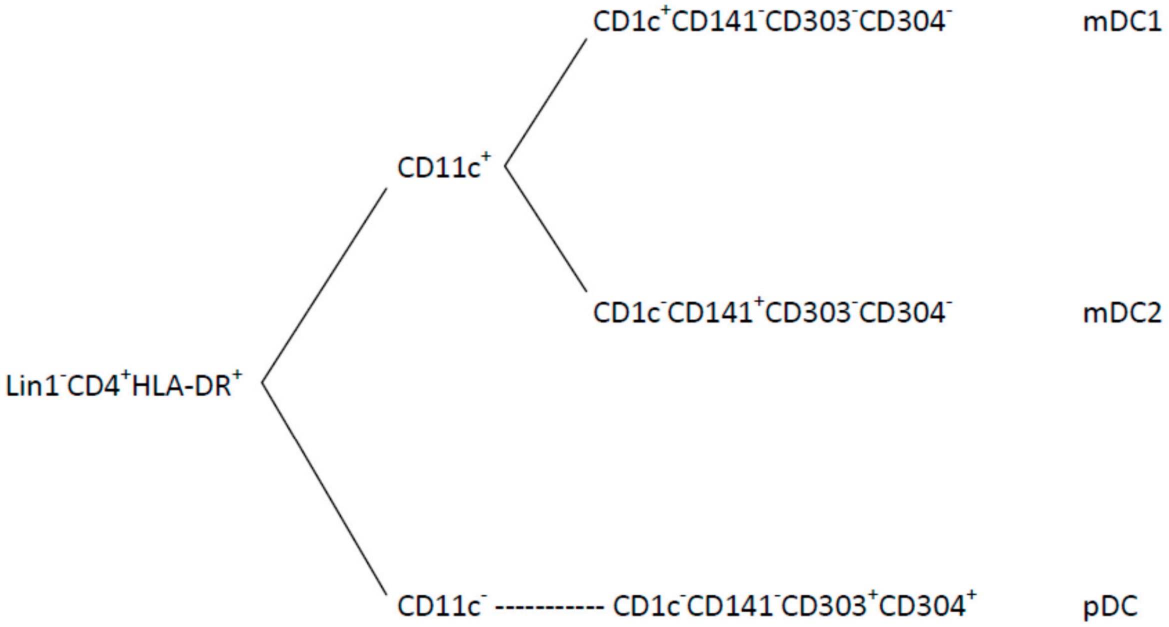

Figure S2. Surface marker expression in different dendritic cell subsets.

8 Isolation of DC Subsets

Dendritic cell subsets were isolated using flow cytometry analysis of cell surface markers. A two-tube method was utilised to differentiate live from dead cells and to separate pDCs from mDCs. This technique was required in order to allow the detection of fluorochromes on particular channels using a flowcytometer (MoFlow XDP, Beckman Coulter Inc., Brea, CA, USA) Using specific antibody combinations. Firstly, PBMCs were incubated for 15 min at 4°C with 1 μl of a viability detection agent (LIVE/DEAD Fixable Dead Cell Stain Kit, Life Technologies, Carlsbad, CA, USA). Cells were then washed in 1 ml of Hank's balanced salt solution before addition of the antibody cocktail. The concentrations of the various antibodies used in the FACS analysis are shown in Table S1. Following addition of the antibodies, the cells were incubated for 30 min at 4°C prior to three washes in Hank's balanced salt solution. Cells were the fixed in 2% formaldehyde prior to analysis.

Table S1. Concentrations of antibodies used in fluorescein-activated cell sorter (FACS) analysis of dendritic cell subsets.

Tube 1	Tube 2	Concentration (μg/ml)
Live/dead fixable dead cell dye	Live/dead fixable dead cell dye	1
Lin-1 FITC	Lin-1 FITC	2.5
HLA-DR ECD	HLA-DR ECD	2.5
CD1c (BDCA-1)- Percp-efluor 710	CD1c (BDCA-1)- Percp-efluor 710	2.5
CD303-PE (BDCA-2)	CD303-PE (BDCA-2)	2.5
CD86-APC	CD197 Alexa 700	1
CD40-APC Cy7	CD80-Pe-Cy7	1
CD11c Pacific Blue	CD83-PeCY5	1
-	CD11c Pacific Blue	1

Lin-1: lineage cocktail-1; FITC: fluorescein isothiocyanate; HLA: human leucocyte antigen

References

1. Dzionek, A.; Fuchs, A.; Schmidt, P.; Cremer, S.; Zysk, M.; Miltenyi, S.; Buck, D.W.; Schmitz, J. BDCA-2, BDCA-3, and BDCA-4: three markers for distinct subsets of dendritic cells in human peripheral blood. *J Immunol* **2000**, *165*, 6037–6046.
2. Sallusto, F.; Lanzavecchia, A. Efficient presentation of soluble antigen by cultured human dendritic cells is maintained by granulocyte/macrophage colony-stimulating factor plus interleukin 4 and downregulated by tumor necrosis factor alpha. *J Exp Med* **1994**, *179*, 1109–1118.
3. MacDonald, K.P.; Munster, D.J.; Clark, G.J.; Dzionek, A.; Schmitz, J.; Hart, D.N. Characterization of human blood dendritic cell subsets. *Blood* **2002**, *100*, 4512–4520, doi:10.1182/blood-2001-11-0097.
